# Supplementary material for: Chemical Synthesis of Trans 8-Methyl-6-Nonenoyl-CoA and Functional Expression Unravel Capsaicin Synthase Activity Encoded by the Pun1 Locus
Source: Molecules. 2022 Oct 13;27(20):6878. doi: 10.3390/molecules27206878 (PMC9606859; doi:10.3390/molecules27206878)
Supplement: Supplementary file 1 [file molecules-27-06878-s001.zip › molecules-1906817-supplementary.pdf]

Chemical synthesis of *trans* 8-methyl-6-nonenoyl-CoA and functional expression unravel capsaicin synthase activity encoded by the *Pun1* locus

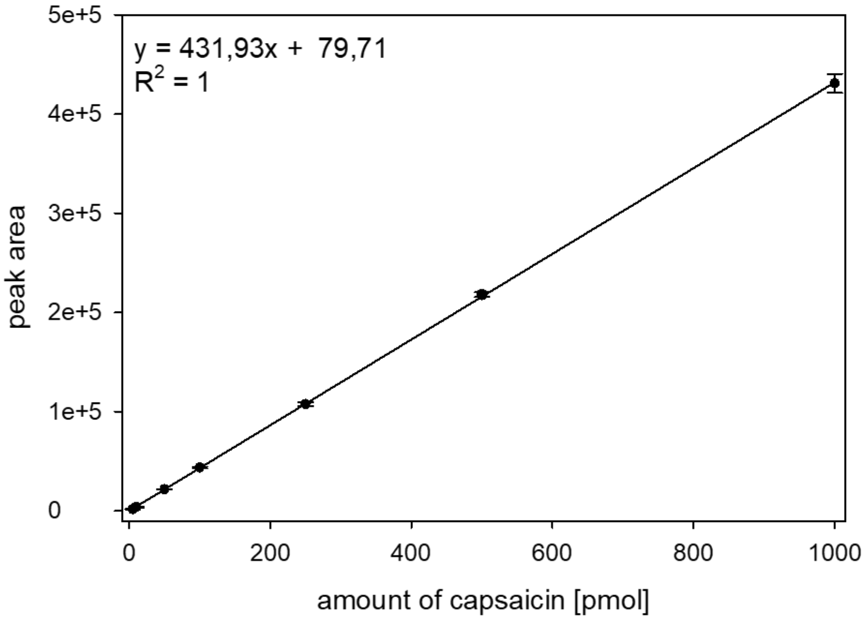

Figure S1. Capsaicin standard curve. Capsaicin concentrations from 1-200 µM dissolved in 100 % MeOH were analyzed by HPLC on a C_{18} Nucleoshell column (5 cm length, 3 mm i.d.) using a water/acetonitrile gradient as described in the Materials and Methods section. Total amounts plotted against peak area at UV_{280nm}. Absolut linearity was observed up to 1 nmol capsaicin (y = 431.93 x + 79.71; R^2 = 1). N= average of three technical replicates.

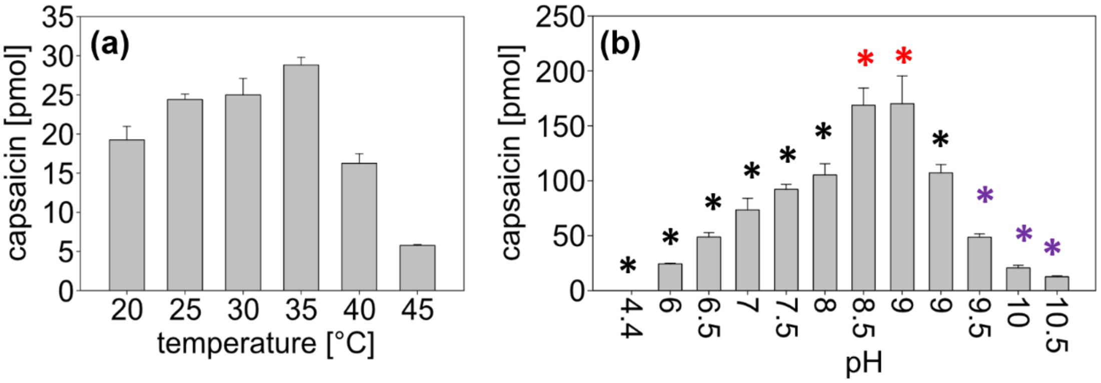

Figure S2. **a**. temperature optimum of partially purified recombinant capsaicin synthase. **b**. pH-optimum of partially purified recombinant capsaicin synthase. black asterisks: potassium phosphate buffer; red asterisks: TRIS/HCl buffer; blue asterisks: sodium carbonate buffer. Each bar is the average of three technical replicates.

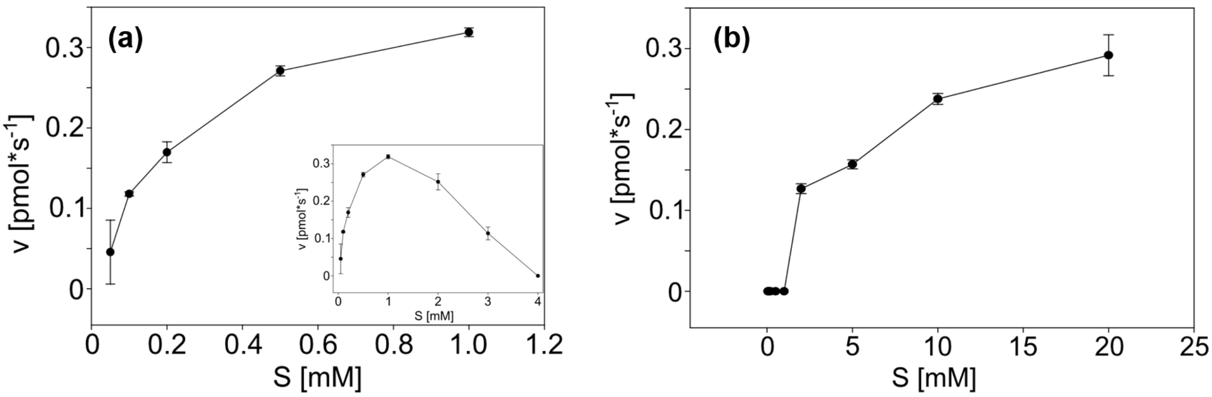

Figure S3. Michaelis-Menten plots of recombinant capsaicin synthase for *trans* 8-methyl-6-nonenoyl-CoA. In the case of the CoA ester the curve fits classical Michaelis-Menten-Kinetics with considerable substrate inhibition above 1 mM *trans* 8-methyl-6-nonenoyl-CoA (a). In the case of vanilloylamine no K_m could be recorded with confidence based on the failure to achieve substrate saturation up to 25 mM (b). Each data point defines the average of three technical replicates.

(a)

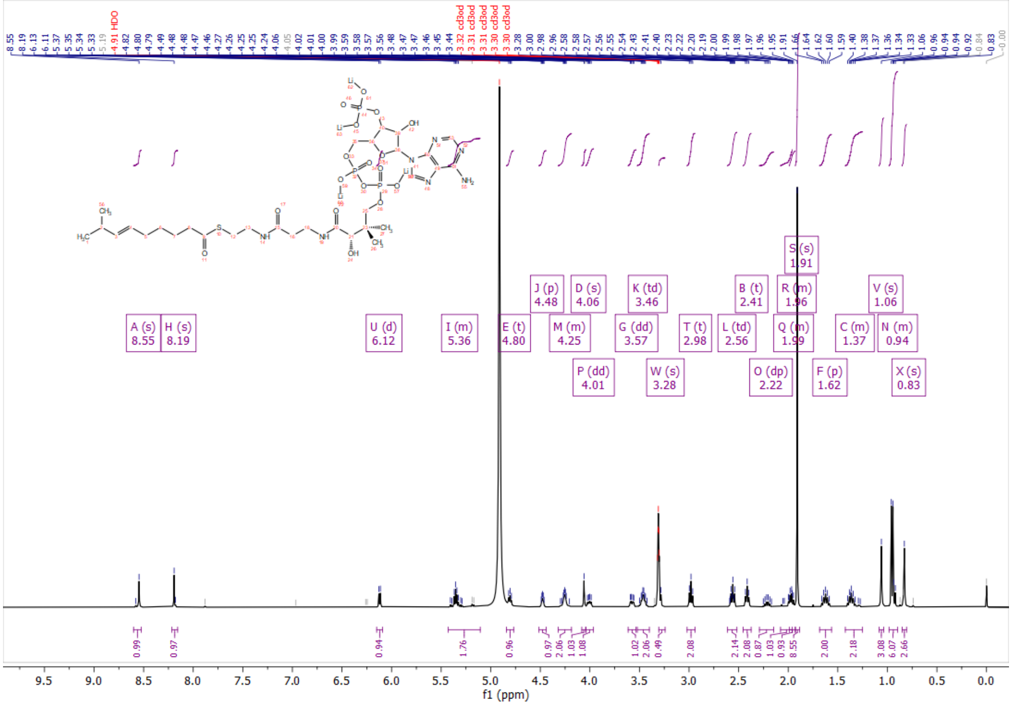

(b)

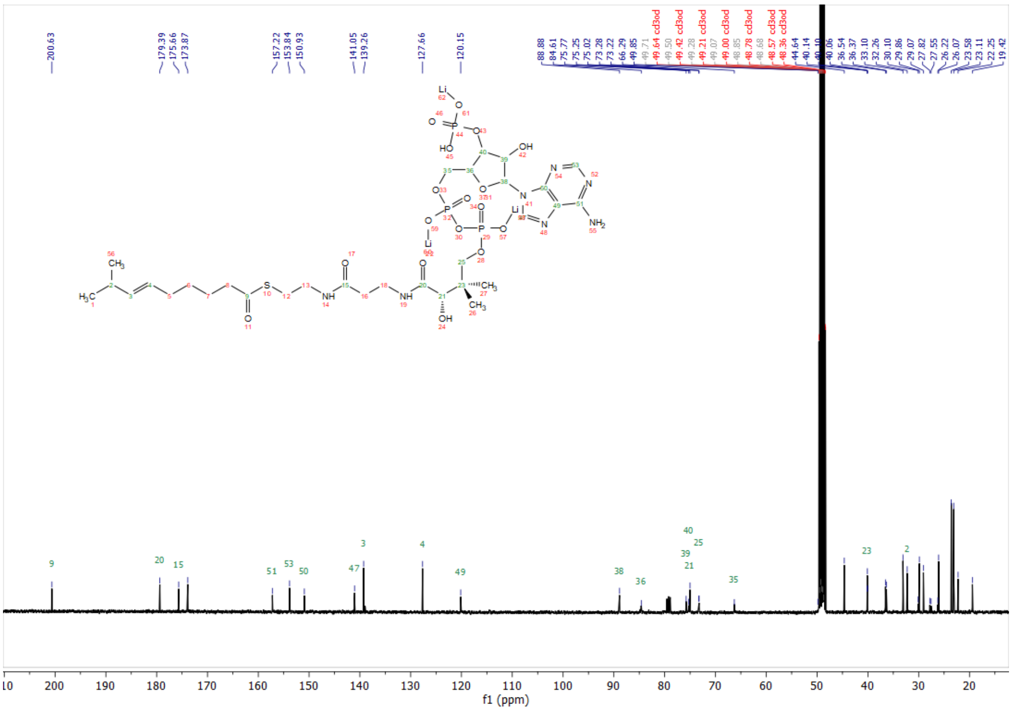

Figure S4. ^{1}H (a) and ^{13}C NMR-signals (b) of synthesized *trans* 8-methyl-6-nonenoyl-ester. The structure of the CoA-ester is shown and proton, respectively carbon signals are marked.

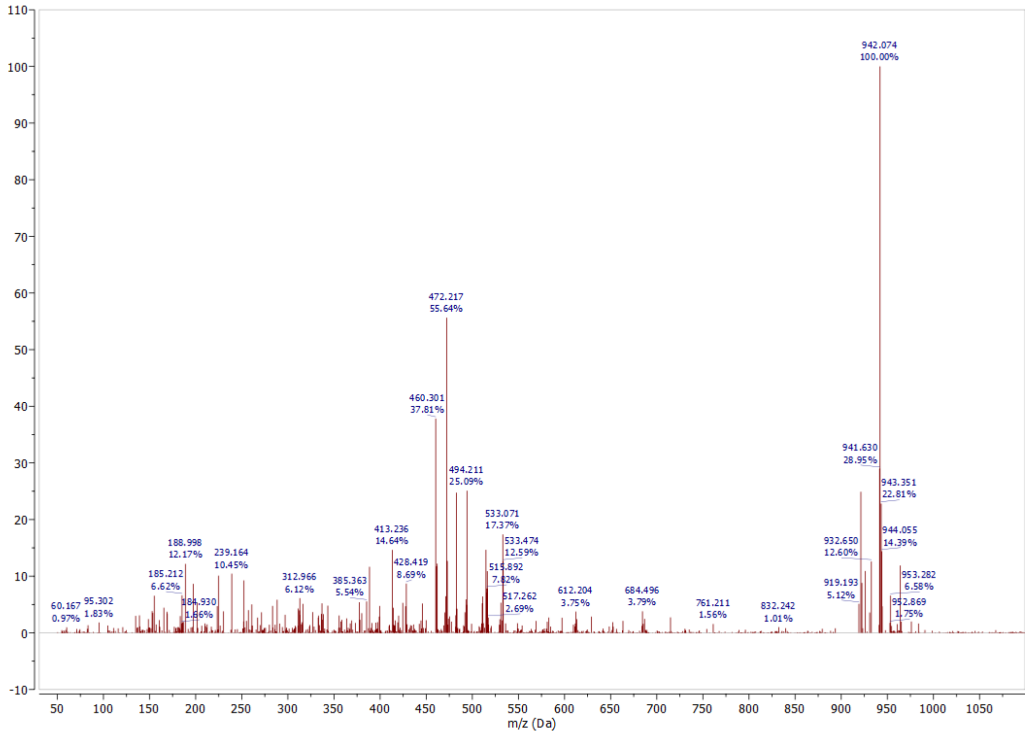

Figure S5. ESI-MS spectrum of *trans* 8-methyl-6-nonenoyl-CoA (API 3200, AB Sciex), negative mode. $C_{31}H_{48}Li_4N_7O_{17}P_3S$ calc. 943.50.

(a)

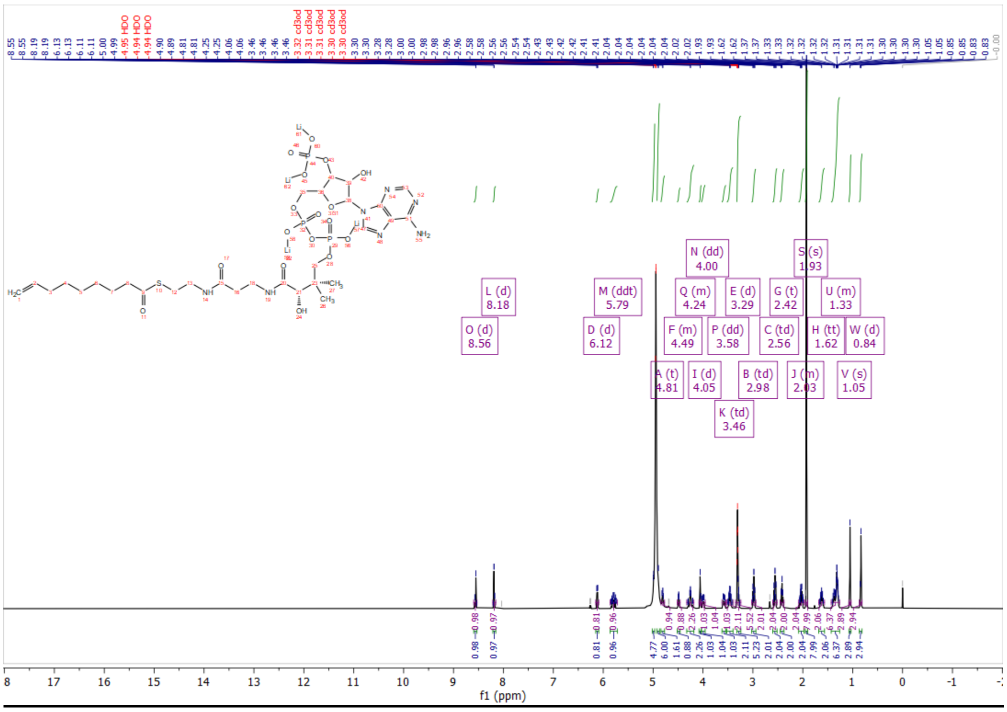

(b)

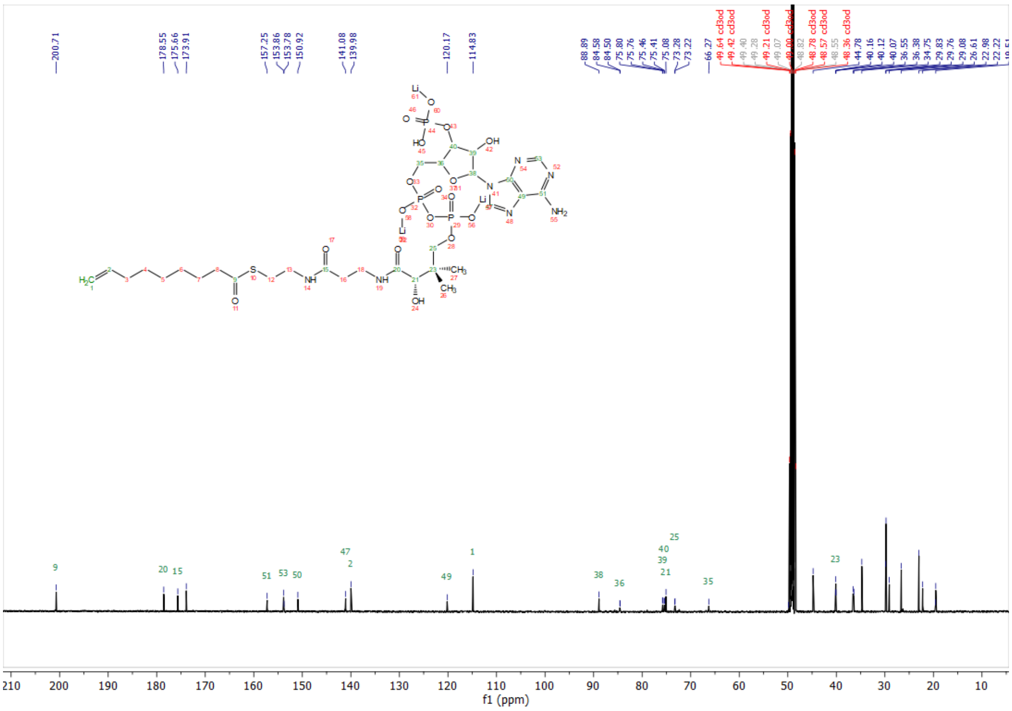

Figure S6. ^{1}H (a) and ^{13}C (b) NMR-signals of synthesized 8-nonenoyl-CoA. Structures of the CoA-ester is displayed. Proton, respectively carbon signals are marked.

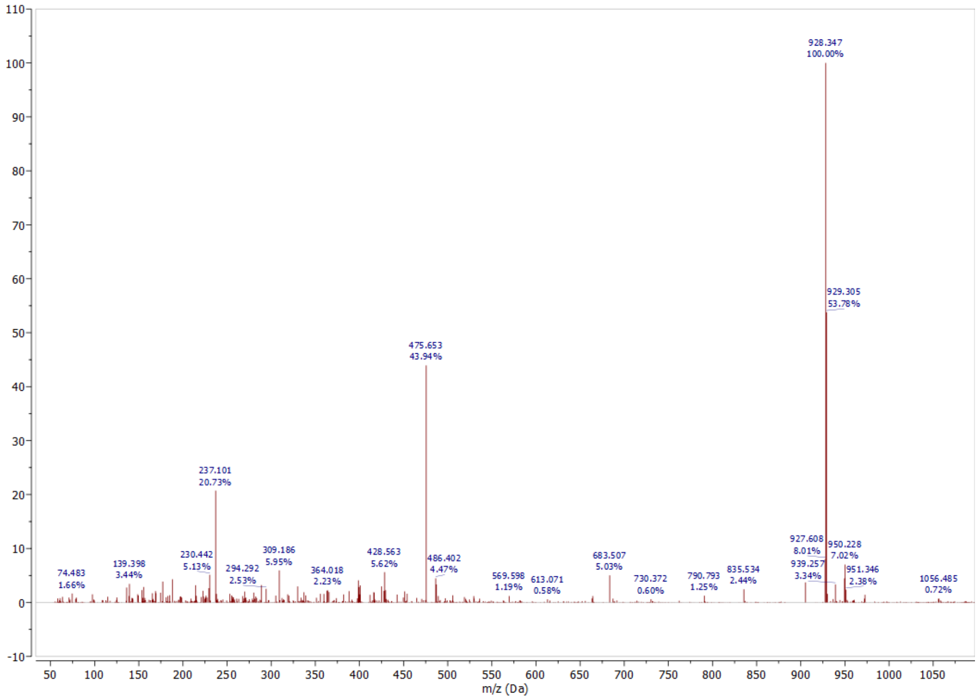

Figure S7. ESI-MS spectrum of 8-nonenoyl CoA (API 3200, AB Sciex), negative mode. $C_{31}H_{48}Li_4N_7O_{17}P_3S$ calc. 929.25.
